# Who Purchases From the Informal Economy and Why?

**DOI:** 10.3389/fpsyg.2022.940076

**Published:** 2022-06-20

**Authors:** Ioana Alexandra Horodnic, Claudia Ioana Ciobanu, Adriana Zaiț, Colin C. Williams

**Affiliations:** ^1^Management, Marketing and Business Administration, Faculty of Economics and Business Administration, Alexandru Ioan Cuza University of Iași, Iași, Romania; ^2^Management BMTM, Faculty of Civil Engineering and Building Services, Gheorghe Asachi Technical University of Iași, Iași, Romania; ^3^Management School, Sheffield University Management School, University of Sheffield, Sheffield, United Kingdom

**Keywords:** consumer behavior, informal economy, rational economic actor, social actor, institutional theory

## Abstract

In recent decades scholars have acknowledged that transactions in the informal economy have not vanished with modernization and industrialization as expected but rather remain an important contemporary aspect of overall production and consumption across the world, in both developing and developed countries. Yet little is known about the profile of the consumers in this realm or what drives them to purchase from the informal economy. A systematic review of the literature investigating consumption in the informal economy reveals a severely underdeveloped area of consumer studies with significant gaps in terms of its theoretical approaches, methods and regional coverage. The findings of the existing literature is that multiple motives are used by consumers for justifying their purchases in the informal economy beyond the dominant simplistic view that they do simply for financial gain or for a lower price (namely, it identifies social ends and failures in formal market provision in terms of availability, speed of provision and quality). The outcome is a recognition that responsibility to reducing this phenomenon with negative effects on governments, businesses, workers and consumers lies not just with public authorities but also practitioners who need to correct the failures in formal market provision. The significant gaps identified in the literature are then used to highlight a comprehensive future research agenda, which includes the need for the development of an institutionalist theoretical perspective when explaining consumers‘ participation in the informal economy and social marketing interventions.

## Introduction

Although the informal economy was historically expected to vanish with industrialization and modernization, empirical studies in the past decade show that the informal economy is still present across both developing and developed countries with negative implications for governments, businesses, workers and consumers. Governments lose tax revenue (Bajada and Schneider, 2005) and have little or no control over the working conditions of those in the informal economy (ILO, 2015). Legitimate businesses face unfair competition from the providers of goods and services in the informal economy (Levy, 2008; Leal Ordóñez, 2014) and customers have little legal recourse when services or goods bought from the informal economy do not meet the health and safety regulations (Williams and Martinez-Perez, 2014a,b). As a consequence, understanding the informal economy is an issue of major interest for multiple stakeholders, including practitioners and policy makers (Williams, 2006a; European Commission, 2016; Williams and Horodnic, 2017; ILO, 2018, 2021; Littlewood et al., 2018).

Currently, around two billion people (i.e., more than 60% of the global workforce) have their main employment in the informal economy (ILO, 2018; Williams and Horodnic, 2019), underlying the importance of understanding this realm since it represents a major sphere of production and consumption globally. Although there are large variations between various regions of the globe, it is important to acknowledge that this phenomenon remains also extensive in developed regions, with an estimated share of 20% of GDP in the OECD countries (Medina and Schneider, 2018) and 18% of the gross value added to the private sector in the European Union (Williams et al., 2017a).

Until now, however, research has focused on the supply side of the informal economy, investigating the socio-demographic characteristics of those working undeclared (Kukk and Staehr, 2014; Putniš and Sauka, 2015; ILO, 2018; Williams and Yang, 2018; Williams and Bezeredi, 2019; World Bank, 2019), what type of work they undertake (Williams, 2014, 2015a; ILO, 2018), estimates of its extensiveness (Williams, 2006b, 2015b, 2017, 2020; Putniš and Sauka, 2015; Williams and Schneider, 2016; OECD, 2017; Medina and Schneider, 2018), and the motives of entrepreneurs starting-up unregistered or continuing to operate in the informal economy (Maloney, 2004; Moris and Polese, 2004; Williams and Schneider, 2016; Shahid et al., 2019; Williams, 2019). Little attention has been given to the demand side of the informal economy in understanding who purchases from the informal economy and consumers' motives for doing so (Williams and Martinez-Perez, 2014a; Littlewood et al., 2018). To omit to study consumer behavior in this extensive market is to ignore a major facet of contemporary consumer culture across the world. Even though informal transactions can be initiated by purchasers who ask ‘how much for the cash?' (Williams and Martinez-Perez, 2014a), Venkatesh and Peñaloza (2006) assert that the informal economy has not so far been evaluated from a marketing perspective, the exception being a study by Arnould (1995). Indeed, Viswanathan et al. (2012) warn that existent literature and theories on consumer behavior are developed using studies of formal markets and therefore, their applicability to informal markets needs to be treated with caution. The aim of this paper, therefore, is to provide a systematic review of what is known about who purchases goods and services from the informal market and their motivations for doing so.

In doing so, this paper advances knowledge on consumer studies in three ways. From a theoretical point of view, we develop a profile of the consumer in the informal economy using previous studies and provide an inventory of the theoretical explanations of consumer motives used to justify their purchase of goods and services from the informal market. Methodologically, this paper provides for the first time a systematic review evaluating the role and the applicability of various explanations of the demand-side of the informal sector. The paper provides a synthesis of all identified quantitative and qualitative studies on the demand side of the informal economy, conducted in both developed and developing countries. Finally, from a practitioner perspective, this paper advances understandings of consumer behavior in the informal sector of major importance for entrepreneurs, competition agencies and policy makers in terms of the actions they need to take to tackle informal sector competition.

To achieve this, the next section describes the methodological approach employed for conducting the literature review. Section Results then presents the findings by providing a profile of the consumer in the informal market, followed by a description of the theoretical explanations used to justify their informal purchasing behavior. This comprises three agency-oriented theoretical perspectives (i.e., rational economic actor, social actor, institutional theory) and the perspective of unintentional or unknowing purchasing. Section Research Gaps and a Future Research Agenda then outlines the current gaps in the literature and the significant avenues for future research. Finally, Section Discussion and Conclusions discuss the implications of the findings for practitioners and for policy makers and provides examples of measures that can be used to tackle informal economy adapted for each theoretical explanation of motivational drivers of consumers.

Before commencing however, the concept of “informal economy” need to be clarified. This realm is known by many different names including the shadow economy, undeclared economy, hidden economy, black economy, cash economy or informal economy (Webb et al., 2013; Williams and Kosta, 2021), generating confusion amongst those not familiar with the field. However, all these terms refer to activities that are unregistered or not declared to the state, for tax, social security and/or labor law reasons. After half a century of academic and political debate, in 2015 the ILO Recommendation 204 was passed which states that “the informal economy does not cover illicit activities,” like drugs, firearms, person traffic or money laundering. These activities belong to the broader criminal economy (ILO, 2021). Therefore, the informal economy refers only to paid transactions of goods and services that are legal in all respects except the fact that they are not declared to the state authorities when they should be declared. This is the consensus definition and the definition here adopted.

## Methodological Approach

### Data Sources

The relevant papers related to consumer behavior in informal markets were identified by conducting a systematic search using the Enformation platform. This platform provides access to the main online bibliographic resources, including: i) indexing services: Science Direct Freedom Collection, Web of Science, Derwent Innovations Index, IEEE/IEL Electronic Library (IEL), Scopus; and ii) publishers: Elsevier, Wiley Journals and Springer. The results were displayed by relevance and we screened the first 100 articles by each key word (i.e., where we had so many displayed). We used the same inclusion criteria for all the databases interrogated. To ensure first-rate standard of evidence, we also screened the references of the selected papers and the personal pages of relevant authors in the informal economy field, to ensure that we did not exclude the papers which might have not appeared in the first search.

The results section emphasizes the role and application of the main identified theories for advancing understanding of the consumer profile and behavior of the informal market.

### Inclusion Criteria

**Figure 1:**
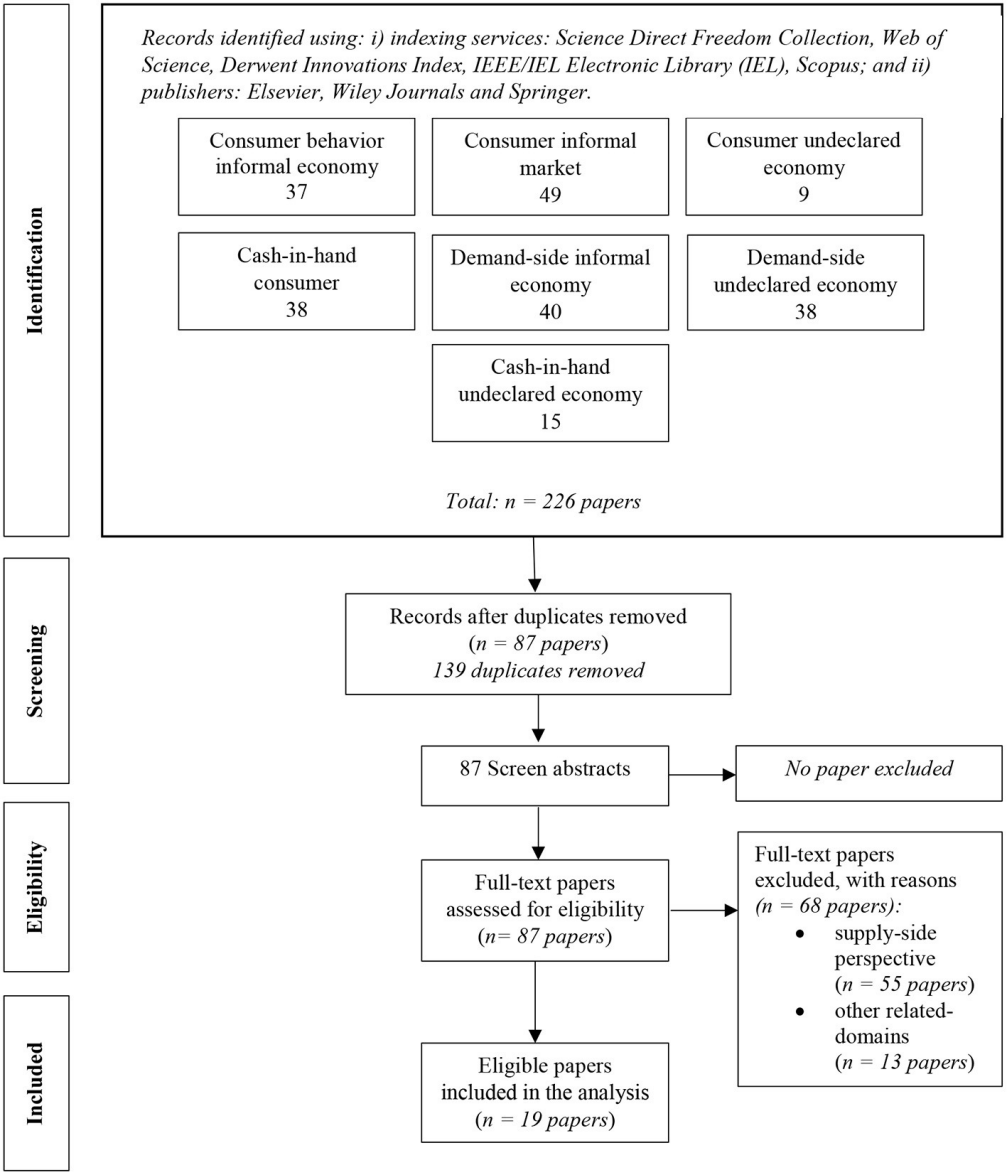
PRISMA flow diagram describing the search results, the screening and study selection.

The inclusion criteria were based on combinations of two important concepts, namely the consumer on the one hand and the informal economy, undeclared economy or their synonyms on the other hand. As such, we used the following keywords “consumer behavior informal economy,” “consumer informal market,” “consumer undeclared economy”, “cash-in-hand consumer,” “cash-in-hand undeclared economy,” “demand-side undeclared economy,” “demand-side informal market.” A limitation of the past 20 years was applied. The publication type included: literature review papers, research articles, conference papers, books, thesis dissertations and official reports. Only papers written in English were kept for further analysis. As PRSIMA flow diagram displays (Figure 1), initially, a total of 226 results were generated for all the key words, out of which 139 were duplicates and removed. The remaining 87 articles were abstract screened, followed by full-text read. These papers have been evaluated by using a custom spreadsheet to record the following: research aim, consumption theoretical model (and/ or the purchasing motive), methodology, sector and sample, in order to increase the rigor of the study (Paul and Criado, 2020). After this operation, 68 records were excluded (55 papers focused on the supply-side perspective and 13 were from other domains than the informal economy). Finally, 19 articles were found eligible and kept in the analysis. Only original research papers which used empirical or theoretical models referring to consumer behavior in informal economy were kept and are displayed in the resultant tables. As such, in sum, the full eligibility criteria included: (i) articles covered by the indexing services and publishers mentioned above; ii) publication date (past 20 years); iii) written in English; iv) empirical or theoretical models referring to consumer behavior in informal economy; v) relevant key word covered. In addition, we have used the AMSTAR checklist provided by Shea et al. (2017) and Mixed Methods Appraisal Tool (MMAT) score sheet by Hong et al. (2018) in order to assess the methodological quality of the undertaken systematic review.

## Results

Before commencing to synthesize the literature, a short description of the data and methodology used in these studies is necessary. The important finding is that a limited number of authors investigate this topic and the empirical studies rely mostly on either small case studies or, if larger scale, on the same databases, namely the three waves of the Eurobarometer surveys on undeclared work (Special Eurobarometer No. 498 from 2019 involving 26,514 respondents; Special Eurobarometer No. 402 from 2013 involving 26,257 respondents and Special Eurobarometer No. 284 from 2007 involving 25,346 respondents). These Eurobarometer surveys (2007, 2013 and 2019) have been used for studying consumer behavior across the European Union or specific European regions such as East-Central Europe where informal markets are more extensive. A second extensive survey conducted in 2015 covers 6,019 respondents from Bulgaria, Croatia and North Macedonia. The remaining studies focus on smaller scale quantitative surveys (i.e., 600 respondents from Lagos, Nigeria, 2017 and 230 households from South Africa, 2015) or qualitative studies (i.e., 3 experimental survey groups from the retail trade in South Africa, 2002; a structured interview on 11 deprived English localities, 1998–2001; a focus group in Zimbabwe, 2006–2008 and 30 interviews in South India). Therefore, in areas such as the United States, Australia and Canada, empirical research on this topic is completely absent.

In nearly all studies, the quantitative analysis employed regression analysis, adapted to the specific variables used, namely: logit regression analysis (Williams and Horodnic, 2016; Williams and Bezeredi, 2017; Littlewood et al., 2018; Williams and Kosta, 2020, 2021), multilevel mixed effects logit regression (Williams and Martinez-Perez, 2014a) or multinomial regression analysis (Williams and Martinez-Perez, 2014b; Williams and Horodnic, 2017; Williams et al., 2017b; Igudia, 2020). The other quantitative studies used descriptive statistics or Principal Component Analysis (PCA) (Tustin, 2004; Marumo and Mabuza, 2018). The results of these empirical studies are briefly synthesized in the next section.

### Who Is the Consumer of Goods and Services Traded on the Informal Market?

A first step is to understand the socio-economic characteristics of the consumer purchasing in the informal economy (Gabbott and Hogg, 1994; Bray, 2008; Littlewood et al., 2018). Reviewing the literature on the demand-side of the informal economy, the effect of various socio-economic characteristics have been evaluated, namely: gender, age, marital status, occupation, education, household size, number of children in the household, area of residence, income, tax morale, trust, perception on the extensiveness of the informal economy and predilection to bargain.

As Table 1 displays, there is great variation in the direction and the effect of the socio-demographic characteristics according to not only the regions investigated but also temporally. Starting with the demographic variables, the results reveal that men are more likely than women to make purchases from informal market, regardless of which European countries are included in the study (Williams, 2008; Williams et al., 2012; Williams and Horodnic, 2016, 2017; Williams and Bezeredi, 2017; Littlewood et al., 2018; Williams and Kosta, 2020, 2021). However, in Nigeria, there is no significant variation between the genders (Igudia, 2020). Meanwhile, in respect to age, there is no clear-cut finding. At the EU27 level or other regions in the European Union, and using data from the 2007 Eurobarometer survey, the finding is that the propensity to purchase undeclared goods and services is higher among younger age groups (Williams, 2008; Williams and Horodnic, 2016). However, this changes over time, with the data from 2019 revealing that older people are more likely to make such purchases, at least if we analyse home repairs and renovations in the European Union (Williams and Kosta, 2020, 2021). A similar result is obtained in a study of 230 households in South Africa (Marumo and Mabuza, 2018). Similarly, different results occur in relation to marital status in different geographical areas. While a study concludes that single people are less likely to buy from informal market (Williams and Horodnic, 2016), another study emphasizes that the subgroup of single people, divorced and separated persons are more inclined to participate to undeclared economic transactions (Williams and Bezeredi, 2017). A wide set of individual occupations that are cash related, including self-employed, managers, other white collars or even manual workers who could have easy access to cash, are significantly correlated with de decision to purchase from the informal market while the unemployed people, students and retired persons are less likely to get involved in informal economy transactions (Williams, 2008; Williams et al., 2012; Williams and Martinez-Perez, 2014b; Williams and Horodnic, 2016, 2017; Williams and Bezeredi, 2017; Littlewood et al., 2018; Williams and Kosta, 2020, 2021). As for education, a study conducted in South-Africa reveal that people with higher education are willing to buy rather from formal market than from informal market (Marumo and Mabuza, 2018). Meanwhile, across EU member states the opposite is the case. People with higher education are more likely to purchase goods and services from the informal economy (Williams, 2008; Williams et al., 2012). Larger households (with more than two members) that are more likely to have financial difficulties get involved in informal transactions in a greater extent than smaller households (Littlewood et al., 2018; Williams and Kosta, 2021). Those who are living in large urban areas are less likely to buy from the informal economy than those who live in rural areas (Williams, 2006a; Williams and Bezeredi, 2017; Williams and Horodnic, 2017; Littlewood et al., 2018; Williams and Kosta, 2020). This might be explained by the trust involved in this type of transactions and access to the informal channels. Both, people with high income and those with financial difficulties make purchases of goods and services from the informal market, the prevalence depending on the regional analyzed area (Williams, 2006a; Williams and Bezeredi, 2017; Littlewood et al., 2018; Marumo and Mabuza, 2018; Williams and Kosta, 2020, 2021).

**Table 1:** Socio-demographic characteristics of the consumer goods and services traded on the informal market.

**Variable**	**Result**	**Region/ Study**
Gender	Men are more likely to make purchases from informal market	• EU27 (Williams, 2008; Williams et al., 2012) • EU28 (Williams and Kosta, 2020) • 11 Central and Eastern European countries (Williams and Horodnic, 2016, 2017; Williams and Kosta, 2021) • Bulgaria, Croatia, North Macedonia (Williams and Bezeredi, 2017; Littlewood et al., 2018)
	No significant difference	• Lagos/Nigeria (Igudia, 2020)
Age	People aged between 25 and 54 are more likely to purchase from the informal market	• EU27 (Williams et al., 2012)
	Young people are more likely to purchase from the informal market	• EU27 (Williams, 2008) • 11 Central and Eastern European countries (Williams and Horodnic, 2016) • Bulgaria, Croatia, North Macedonia (Williams and Bezeredi, 2017)
	Older people are more likely to purchase from the informal market	• EU28 (Williams and Kosta, 2020) • 11 Central and Eastern European countries (Williams and Kosta, 2021) • 230 households from South Africa (Marumo and Mabuza, 2018)
	No significant difference	• EU27 (Williams and Martinez-Perez, 2014b) • Bulgaria, Croatia, North Macedonia (Williams and Bezeredi, 2017) • Lagos/Nigeria (Igudia, 2020)
Marital status	Single people are less likely to purchase from the informal market than married/cohabiting ones	• 11 Central and Eastern European countries (Williams and Horodnic, 2016)
	Divorced and separated people are more likely to purchase from the informal market	• Bulgaria, Croatia, North Macedonia (Williams and Bezeredi, 2017)
Occupation	Self-employed workers are more likely to purchase from the informal market	• EU27 (Williams, 2008; Williams et al., 2012; Williams and Martinez-Perez, 2014b) • 11 Central and Eastern European countries (Williams and Horodnic, 2016, 2017; Williams and Kosta, 2021) • Bulgaria, Croatia, North Macedonia (Williams and Bezeredi, 2017)
	Managers are more likely to purchase from the informal market	• EU27 (Williams, 2008; Williams et al., 2012) • 11 Central and Eastern European countries (Williams and Horodnic, 2016, 2017)
	Other white collar workers are more likely to purchase from the informal market	• EU27 (Williams, 2008; Williams et al., 2012) • 11 Central and Eastern European countries (Williams and Horodnic, 2017)
	Manual workers are more likely to purchase from the informal market	• 11 Central and Eastern European countries (Williams and Horodnic, 2017)
	Retired, students (unemployed) are less likely to purchase from informal market	• EU28 (Williams and Kosta, 2020) • Bulgaria, Croatia, North Macedonia (Littlewood et al., 2018)
Education	People with higher education are more likely to purchase from the informal market	• EU27 (Williams, 2008; Williams et al., 2012)
	People with higher education are less likely to purchase from the informal market	• South Africa (Marumo and Mabuza, 2018)
	No significant difference	• EU28 (Williams and Kosta, 2020)
Household size	Those living in households with 2 or more people are more likely to purchase from the informal market than those with one person	• 11 Central and Eastern European countries (Williams and Kosta, 2021) Bulgaria, Croatia, North Macedonia (Littlewood et al., 2018)
	No significant difference	• 11 Central and Eastern European countries (Williams and Horodnic, 2016)
Number of children in the household	No significant difference	• 11 Central and Eastern European countries (Williams and Horodnic, 2016)
Area of residence	People from urban/large towns are less likely to purchase from the informal market	• EU28 (Williams and Kosta, 2020) • 11 Central and Eastern European countries (Williams and Horodnic, 2017) • Bulgaria, Croatia, North Macedonia (Williams and Bezeredi, 2017; Littlewood et al., 2018) English localities (Williams, 2006a)
	No significant difference	• 11 Central and Eastern European countries (Williams and Horodnic, 2016)
Income	People with high income are more likely to purchase from the informal market	• Bulgaria, Croatia, North Macedonia (Williams and Bezeredi, 2017; Littlewood et al., 2018) • English localities (Williams, 2006a)
	Wealth households are less likely to purchase from the informal market	• South Africa, (Marumo and Mabuza, 2018)
	People having difficulties in paying bills is more likely to purchase from the informal market	• •EU28 (Williams and Kosta, 2020) 11 Central and Eastern European countries (Williams and Kosta, 2021)
	No significant difference	• 11 Central and Eastern European countries (Williams and Horodnic, 2016)
Tax morale	Consumers with low tax morale are more likely to purchase from the informal market	• EU27 (Williams and Martinez-Perez, 2014a) EU28 (Williams et al., 2017b) • 11 Central and Eastern European countries (Williams and Horodnic, 2016, 2017) • Bulgaria, Croatia, North Macedonia (Williams and Bezeredi, 2017; Littlewood et al., 2018) • Slovenia (Culiberg and Bajde, 2013)
Trust	People with lower horizontal trust are more likely to purchase from the informal market	• Chennai/South India (Viswanathan et al., 2012) • Bulgaria, Croatia, North Macedonia (Williams and Bezeredi, 2017)
Perception on the extensiveness of the informal economy	People who perceive that 50% of more of the people in the society they live in are suppliers in the informal economy are more likely to purchase from the informal economy	• Bulgaria, Croatia, North Macedonia (Williams and Bezeredi, 2017)
Predilection to bargain	Households that prefer to bargain are more likely to purchase from the informal market	• South Africa (Marumo and Mabuza, 2018) • Zimbabwe (Chikweche and Fletcher, 2010)

Moving to attitudinal variables, the finding is that tax morale affects the consumer propensity to purchase undeclared goods and services (Culiberg and Bajde, 2013; Williams and Martinez-Perez, 2014a; Williams and Horodnic, 2016, 2017; Williams and Bezeredi, 2017; Williams et al., 2017b; Littlewood et al., 2018). This holds valid regardless of the analyzed regional area or the time frame, suggesting that it plays a major role in determining whether or not to make purchases from the informal market. Similarly, the level of the horizontal trust (between the citizens) is directly associated with the decision to purchase from informal market. These variables have been frequently analyzed in studies of the supply-side of the informal economy but in just two studies of the demand-side of the informal market (Viswanathan et al., 2012; Williams and Bezeredi, 2017). The finding is that the lower the level of trust, the higher the probability to make purchases from the informal economy. Another variable, measuring the horizontal trust, namely the perception on the extensiveness of the informal economy provides similar results (Williams and Bezeredi, 2017). Finally, a qualitative study reveals that the customer behavior on the market can be explained by the predilection to bargain, households that prefer to bargain being more likely to purchase from the informal market (Chikweche and Fletcher, 2010; Marumo and Mabuza, 2018).

The outcome of this review is a call for a more nuanced understanding of the profile of the consumer of goods and services from the informal economy. What is valid for some regions or sectors is not valid for other ones. As such, entrepreneurs, competition agencies and policy makers need to tailor their approaches toward tackling the informal market to fit the consumer profile valid in the area they operate in.

### Which Are the Motivational Drivers Pushing the Consumer to Informal Economy? A Synthesis of the Theoretical Perspectives

The review of the previous empirical research underlined three agency-oriented theoretical perspectives (i.e., rational economic actor, social actor, institutional theory) and the perspective of unintentional or unknowingly purchase. Most of the papers investigate these theories together as complementary explanations and not exclusive explanations for purchaser behavior. A synthesis of these papers, presented in chronological order and including the sample, region, sector, method, findings and theoretical perspectives employed is presented in Table 2. The next section discusses in depth the findings.

**Table 2:** Theoretical explanations for the consumer behavior in the informal economy.

**References**	**Sample, region and time frame**	**Sector**	**Methodology**	**Purchasing motives or consumption model**	**Theoretical explanations/ Concepts**
Tustin (2004)	• 3 experimental survey groups • South Africa • 1980–2003 secondary data • 2001–2002 experimental data	Retail trade	Quantitative	• Composition of the informal retail trade product basket influenced by affluence, level, demographics, taste and life style	• Rational economic actor (i.e., consumer spending patterns)
Williams (2006a)	• 861 face-to-face interviews • 11 English localities • 1998–2001	Household service sector	Quantitative	• Lower price: 31% • Help the supplier financially: 22% • Community building: 47%	• Rational economic actor • Social actor
Williams (2008)	• 26,659 face-to-face interviews • 27 EU Member States • Eurobarometer survey 2007	All	Quantitative	• Lower price: 31% • Social motives: 24% • Formal market provision failures: 39%	• Rational economic actor • Social actor • Institutional theory
Chikweche and Fletcher (2010)	• Focus groups • Zimbabwe • 2006–2008	All/ Subsistence markets	Qualitative	• Psychological needs: 93% • Uncertainty of product availability: 93% • Price: 93% • Peer and social network: 81% • Family: 78% • New cheaper and performant products: 64% • Firms' promotion activities: 58% • Environmental hazards: 54% • Convenience: 53%	• Rational economic actor • Social actor Institutional theory
Williams et al. (2012)	• 26,659 face-to-face interviews • 27 EU states • Eurobarometer survey 2007	Property and construction sector	Quantitative	• Lower price: 38% • Social motives: 8% • Formal market provision failures: 14%	• Rational economic actor • Social actor • Institutional theory
Viswanathan et al. (2012)	• 30 interviews • Chennai/South India • 2008–2012	All/Subsistence Markets	Qualitative	• Marketplace environment: *interactional empathy* and *enduring relationships* • Marketplace exchange: responsiveness, fluid transactions, constant customization	• Social actor
Culiberg and Bajde (2013)	• 367 respondents • Slovenia 2013	All	Qualitative, Quantitative	• Model of consumer ethical decision: moral dimensions, relativism, idealism	• Institutional Theory (i.e., tax morale)
London et al. (2014)	• 555 respondents • rural India • 2008–2009	All	Quantitative	• Factors influencing purchasing decision: wellbeing (economic, relationship, capability) and strength of formal institutional environment	• Institutional Theory
Williams and Martinez-Perez (2014a)	• 26,659 face-to-face interviews • 27 EU states • Eurobarometer survey 2007	All	Quantitative	• Lower price: 44% • Social motives: 10% • Formal market provision failures: 15%	• Rational economic actor • Social actor • Institutional theory
Williams and Martinez-Perez (2014b)	• 26,659 face-to-face interviews 27 • EU states • Eurobarometer survey 2007	All	Quantitative	• Lower price: 44% • Social motives: 10% • Formal market provision failures: 15%	• Rational economic actor Social actor Institutional theory
Williams and Horodnic (2016)	• 11,131 face-to-face • structured interviews 11 Central and • Eastern European countries Eurobarometer survey 2013	All	Quantitative	• Lower price: 30% • Social motives: 26% • Formal market provision failures: 17%	• Rational economic actor • Social actor • Institutional theory
Williams and Bezeredi (2017)	• 6,019 face-to-face interviews, • Bulgaria, Croatia, • North Macedonia 2015	All	Quantitative	• Lower price: 57,1% • Social motives: 25,1% • Formal market provision failures: 48,9%	• Rational economic actor • Social actor • Institutional theory
Williams et al. (2017b)	• 27,563 face-to-face interviews • EU28 Eurobarometer survey 2013	All	Quantitative	• Lower price: 30% • Social motives: 13% • Formal market provision failures: 11%	• Rational economic actor • Social actor • Institutional theory
Littlewood et al. (2018)	• Focus groups and • 6,019 face-to-face interviews • Bulgaria, Croatia, North Macedonia- 2015	All	Qualitative, Quantitative	• Lower price: 52,5% • Social motives: 17,5% • Formal market provision failures: 48,9%	• Rational economic actor • Social actor • Institutional theory
Marumo and Mabuza (2018)	• 230 household surveys • South Africa 2015	Vegetable market	Quantitative	• Factors with positive influence: low price, age, education, bargain/ convenience • Factors with negative influence: wealth, food safety and quality	• Rational economic actor
Tang et al. (2020)	• 2,585 individuals urban • China 2012–2012	Street vending	Qualitative, Quantitative	• Consumer types identified**:** Conservative, Balanced, Frustrated, and Adventurous. For all types of behavior explained by financial, social and institutional factors	• Rational economic actor • Social actor • Institutional theory
Williams and Kosta (2020)	• 27,565 face-to-face interviews • EU28 Eurobarometer survey 2019	Home repairs and renovation sector	Quantitative	• Lower price: 25,1% • Social motives: 13,8% • Formal market provision failures: 8,1 % • Unintentional: 10.4%	• Rational economic actor • Social actor Institutional theory • Unintentional purchase
Igudia (2020)	• 600 respondents Lagos/Nigeria 2017	Street Vending	Quantitative	• Social redistributive rationales: 9,9% • Financial gain: 12,5% • Formal market provision failures: 53,3% • Multifeature (i.e., survival, quick/reliable money, easy sales): 24,3%	• Rational economic actor • Social actor • Institutional theory
Williams and Kosta (2021)	• 11,171 face-to-face interviews • 11 Central and Eastern • European countries • Eurobarometer survey 2019	Home repair and renovation sector	Quantitative	• Lower price: 20,1% • Social motives: 12,8% • Formal provision failure: 12,8 % • Unintentional: 3,6%	• Rational economic actor • Social actor Institutional theory • Unintentional purchase

#### Rational Economic Actor Theoretical Perspective

The dominant view when explaining the informal economy assumes that individuals are rational actors taking decisions that enable them to maximize their financial benefits. This theory, rooted historically in the work of Jeremy Bentham (1788), has been developed and applied to informal economy by Allingham and Sandmo (1972). According to this view, individuals engage in illegal activities such as participating in the informal economy either as suppliers if the benefits are higher than the risks associated with this activity. This perspective has been widely tested on the supply-side of the informal economy and was considered for a long time the predominant explanation for participation in the informal economy (Williams, 2006a, 2008). Previous results show that this cost/price explanation is more prevalent in less developed regions where workers engage in the informal economy as a survival strategy rather than as a matter of choice like in developed countries (Ketchen et al., 2014; Williams, 2017). As such, the rational economic actor perspective adopts a marginalization thesis when analyzing developing countries or deprived groups viewing bottom-of-the-pyramid (BOP) informal markets as marginal subsistence markets for marginalized workers (De Soto, 1989; Tustin, 2004; La Porta and Shleifer, 2014; Williams and Martinez-Perez, 2014a; Meagher, 2015; Williams, 2017; Williams and Kosta, 2021).

Applied to the consumer perspective, this rational economic actor explanation can be viewed as consumers taking the simple opportunity to obtain the cheapest possible price, with consumers sometimes even initiating such purchases by asking providers “how much for cash?” (Schneider and Enste, 2000; Williams, 2008). As such, from the consumer perspective, each transaction implies a quick evaluation of the cost/benefit ratio and if the gain for the customer is higher in the informal market, where the transaction is not subject to fiscal burdens (i.e., VAT or income tax), then the decision is made in favor of informal market purchase (Williams, 2017, 2019).

Previous literature on subsistence markets, where populations are poor and live in deprived regions, validates this theoretical perspective and show that consumers use the informal economy, such as bottom-of-the-pyramid (BOP) markets, to obtain a lower price, meaning that they obtain greater utility from their limited monetary resources (Tustin, 2004; Chikweche and Fletcher, 2010; World Bank, 2019; Igudia, 2020).

In more developed countries, such as the European countries, the lower price explanation represents only one of several reasons for purchasing from the informal economy. For example, studies using survey data from 2007, show that 44% of the European consumers of the goods and services from the informal market justified their decision to purchase from the informal market due to a lower price alone and for other 28% of the customers the lower price was one of the several motives mentioned (Williams and Martinez-Perez, 2014a,b). Later, in 2013, studies of the European Union show that the monetary motive decreased in importance in the purchase process. The lower cost explanation was present as the sole reason for just 30% of purchases and in 31% of purchases it was one of the several reasons driving the consumer to the informal market (Williams et al., 2017b).

Other research, on smaller regions, provide similar results. Lower price does not represent the only valid explanation for consumers engaging in the informal economy. For example, a study across 11 Central and Eastern European Countries indicate a prevalence of the lower cost justification alone in 30% of informal purchases and as one of the explanations given in an additional 31% of cases (Williams and Horodnic, 2016). Meanwhile, a study investigating consumers in Bulgaria, Croatia and North Macedonia display that the lower price motivation alone was valid for 57% of informal purchases (Williams and Bezeredi, 2017).

Finally, a more recent study using data from a 2019 survey and focusing on the home repairs and renovation sector show a decline in the prevalence of the financial gain motive alone. Consumers of goods and services in this sector explain their purchases as being due to a lower price in just 25% of cases (Williams and Kosta, 2020) whilst in 2007 the figure was 38% (Williams et al., 2012).

As such, the finding is that lower price alone is not the only reason or the main reason for consumers choosing to purchase from the informal economy. There are other motives involved such as the failures of the formal market provision or the aim of pursuing social ends. This finding is important for practitioners and competition agencies who, instead of focusing on the dominant idea of unfair competition from the informal market in terms of price, could seek to understand how to adapt their services and products in such a manner that would attract consumers from the informal market to their services and products. This would enable not only an increase in the market share and the number of customers for their companies but also, by joining with public authorities in tackling the informal economy, they would contribute to wider societal goals. It can therefore be seen as an additional arm of their corporate social responsibility (CSR) strategies.

#### Social Actor Theoretical Perspective

Over the past decade or so, the view of consumers as rational economic actors whose behavior is governed by financial gain has been transcended. The social actor theoretical perspective criticizes the narrow view of economic endeavor as always profit-driven behavior and analyses the complex nature of exchanges, recognizing multiple other rationales for which consumers might engage in informal market, including social motives which prevail over the monetary justification (Zelinzer, 1994, 2005; Escobar, 1995; Bourdieu, 2002; Gibson-Graham, 2006; Williams, 2008; Kaze et al., 2011; Williams and Kosta, 2020). Indeed, cementing social relationships or building relationships and therefore building social capital is considered by some researchers the main reason behind informal activities (Zelinzer, 1994, 2005).

Adopting this lens, a few studies evaluated this social actor theory in relation to the demand-side of the informal economy (Williams, 2006a, 2008; Chikweche and Fletcher, 2010; Viswanathan et al., 2012; Williams et al., 2012; Williams and Martinez-Perez, 2014a,b; Williams and Horodnic, 2016; Littlewood et al., 2018; Marumo and Mabuza, 2018). The finding is that a significant number of transactions of goods and services in the informal economy involve kin, acquaintances, neighbors, friends or work colleagues. For example, one might pay close social relations for goods or services (i.e., babysitting, house cleaning) to consolidate the relationship or to help them if in need of money, as providing money for a service does not conjure up any notion of charity, which might lead them to refuse the money (Kempson, 1996). Therefore, from this perspective, informal transactions are seen as a form of community exchange or active citizenship grounded in notions of mutual aid and reciprocity (Williams, 2008).

This social actor theory perspective was employed to analyse the demand side of the informal economy for the first time by Williams (2008) using data from a Eurobarometer survey conducted in 2007. The findings show that other reasons than financial gain justify why consumers get involved in undeclared transactions for one third of purchases, out of which pursuing social ends account for 25% of all purchases (Williams, 2008). Therefore, consumers choose to buy products or services from the informal market in order to strengthen their social relations. Doing favors for friends, kin and neighbors (14%) or helping friends in need to earn some money (11%) are also justifications for purchasing from informal market. A consequent study conducted in the EU28 using the 2013 Eurobarometer survey data concludes that social motives alone explain 13% of the purchases and, in addition, represent one of multiple reasons consumers purchase in the informal market in 23% of cases (Williams et al., 2017b). Similarly, another quantitative study from 2017 indicates that social redistributive rationales explain informal street vending market transactions in Nigeria in 9.9% of cases (Igudia, 2020). Examining repair and renovation services, an analysis of the 2007 Eurobarometer survey finds that the social theoretical explanation holds for 8% of transactions (Williams et al., 2012) and 13.8% of such transactions in the 2019 Eurobarometer survey (Williams and Kosta, 2020), displaying its growing importance as an explanation.

Qualitative studies also reveal social actor explanations as a driver of the consumer decision to purchase informally (Chikweche and Fletcher, 2010; Viswanathan et al., 2012). Countries such as India or Zimbabwe have unique marketing particularities and encounter high prevalence of informal transactions. Previous research results indicate that community building, peer and social network, family and social contract as factors influencing the purchase in subsistence BOP markets. Indeed, informal purchases occur in village markets and local shops and the consumers compare the prices across the sellers and negotiate. Therefore, the social actor theoretical explanation is valid in various regional areas and for different type of consumer, and proves to be a direct challenge to the limited explanation of a lower price.

#### Institutional Theoretical Perspective

Another explanation is that consumer purchasing in the informal economy is driven by the institutions. Institutions are the rules of the game in a society. According to a variant of institutional theory (North, 1990; Helmke and Levitsky, 2004) applied to explain participation in the informal economy, each society has both formal institutions describing the laws and regulations, as well as informal institutions describing the norms, beliefs and values of citizens and consumers about the acceptability of different behaviors (North, 1990; Gibson-Graham, 2006). Employing this institutionalist lens, the informal economy falls outside the regulations of formal institutions but within the acceptable behavior defined by the norms, values and beliefs of the informal institutions (Williams, 2017). This perspective has been intensively used to explain the supply-side of the informal economy and the previous findings can be summarized in three waves of thought in institutional theory. In the first wave of thought, participation in the informal economy was explained by the shortcomings of the formal institutions (i.e., resource misallocations and inefficiencies, voids and weaknesses, powerlessness and instability and uncertainty). The second wave started to recognize the role of informal institutions and argued that even if formal institutions have shortcomings, participation in informal economy would not happen if it would not be seen as an acceptable behavior by the informal institutions. Therefore, participation in informal economy appears when there is an asymmetry between the two types of institution. Finally, the third wave of thought put the first two waves of thought together and argued that the shortcomings of the formal institutions produce an asymmetry between the formal and informal institutions (Williams, 2017). Williams and Horodnic (2020) provide an extensive review of the findings for the supply-side of the informal economy, underlying the specific drivers of the informal economy, analyzing both, drivers related with formal institutions (e.g., level of corruption, modernization of government, income inequalities) and drivers related with informal institutions (e.g., the level of trust that citizens display toward their government, the level of trust in their peer citizens in behaving in a compliant manner).

Turning to the demand-side of the informal economy, and starting with the first wave, previous research shows that the failures of the formal economy in delivering goods and services to citizens (i.e., the lack of availability, speed and quality of the formal economy) motivate the consumer to purchase from the informal economy (Williams, 2008; Williams and Horodnic, 2016; Williams and Bezeredi, 2019). To exemplify this, it was found that customers choose to buy informal goods and services due to formal provision being poorer in terms of speed of provision (21% of the consumers), quality of provision (8% of the consumers) or even due to the lack of availability of the wanted good or service on the formal market (10% of consumers) (Williams, 2008). In terms of the second wave of thought and starting to explore the role of informal institutions and the asymmetry between formal and informal institutions, little research has been conducted. No study so far has investigated whether the trust in formal institutions (i.e., vertical trust) influences the consumer decision to participate to the informal economy. However, the influence of horizontal trust or the trust that other members of society act in a legal and responsible manner, has been investigated and it was found to have a direct influence on the purchaser decision to participate to the informal economy. A study by Williams and Bezeredi (2017) in Bulgaria, Croatia and North Macedonia, shows that the likelihood to purchase from the informal economy is higher when the level of horizontal trust is lower. As such, when the consumers perceive that other consumers make purchases of undeclared goods and services, they are more likely to engage in a similar behavior. The role of the asymmetry between formal and informal institutions in the purchasing decision was investigated in a slightly higher number of studies using the tax morale as a proxy. The finding is that the lower the tax morale, the higher the probability that a consumer will choose informal markets over formal ones. This holds valid for various time frames and regions (Williams and Martinez-Perez, 2014a; Williams and Horodnic, 2016, 2017; Williams and Bezeredi, 2017; Williams et al., 2017b; Littlewood et al., 2018). Similarly, a low level of tax morale has been identified as an essential feature of the informal market customer profile (Culiberg and Bajde, 2013). Nevertheless, a deeper investigation capturing the internalized and extrinsic features of tax morale has been conducted so far only for investigating the supply side of the informal economy (Onu et al., 2019).

Finally, no previous study on the demand-side of the informal economy (i.e., the consumers) employed the third wave of thought seeking to analyse the role of formal institutions in producing the asymmetry between formal and informal institutions, despite the extensive results that support this theory on the supply side of the informal economy. This opens up valuable directions for future research which will be returned to in the next section.

#### Unintentional Purchase Theoretical Perspective

While the previous three theoretical perspectives imply that the consumer is aware that they participate in the informal market and do this as a voluntary choice, it cannot be asserted that all purchases from the informal economy are made knowingly by consumers. Similar to the supply-side of the informal economy when workers do not meet the regulations related to income tax, social contribution or labor law because they might not be up to date on what regulations are in place (Ricardson, 2006), the consumer might also participate in the informal economy because they are unaware that their transaction is informal until after the purchase has been made and they did not receive a receipt or an invoice. This unintentional facet has been recently introduced as a reason customers give for justifying their involvement in the informal market. The finding is that in the home repairs and renovation sector, about 4 to 10% of the purchases, depending on the analyzed regional area, are made purely unintentionally, the consumer realizing only after the purchase has been made (Williams and Kosta, 2020, 2021).

In sum, to better understand consumer behavior in the informal economy, it is important to consider both intentional purchases (i.e., rational economic actor, social actor and institutional theoretical perspectives) as well as unintentional purchases. However, as the results of this review show, previous research on consumers in the informal economy is scarce despite the high importance for practitioners, competition agencies and governments which will be detailed in more detail in the next section.

## Research Gaps and a Future Research Agenda

This review of the existent literature on the consumer purchases from the informal economy has revealed that the topic is severely underdeveloped. The little knowledge available so far is developed by a narrow number of researchers and using mainly the same large-scale studies. The small-scale studies are also limited. Indeed, there are regional areas where the topic is completely untouched (i.e., the United States, Australia, and Canada). In terms of theory, unlike the extensive research conducted to explain why people work in the informal economy (the supply-side), a limited number of theories found valid when explaining the supply-side have been applied and tested for investigating the consumer of these informal goods and services. For example, consumers' tax morale proved valid in explaining their participation in the informal economy regardless of the type of customer or geographical area (as Table 1 displayed). However, it is surprising that no study has sought to understand what determines the level of tax morale of customers and what creates the asymmetry between the formal and informal institutions (i.e., the third wave of thought in the institutional theory for explaining participation in informal economy). As such, for developing the theory on consumer behavior in informal markets and drawing inspiration from the extensive research and results on suppliers' behavior, we propose a novel multidisciplinary approach deploying an institutional theory perspective.

Drawing upon a neo-institutional theoretical perspective, future research needs to recognize that consumers‘ behavior is shaped by the institutional environment they are embedded in, defined by three pillars namely, the regulative, normative and cultural-cognitive pillars (Scott, 2008). The regulatory pillar prescribes the formal institutions and refers to the formal laws and regulations which encourage certain behaviors and discourage or sanction other behaviors (i.e., formal rules related to the obligation of issuing receipts when selling goods and services, meeting the health and safety regulations and so forth). The normative pillar refers to the norms and values existent in a society, defining what is considered as appropriate and acceptable behavior (i.e., attitudes toward purchasing from the informal sector). The cultural-cognitive pillar refers to how some behaviors are taken for granted based on culturally supported common shared understandings and beliefs (Scott, 2008; Williams, 2017). This relates here to how informal activities are undertaken unthinkingly, as for example, routine purchases from informal vendors or how people do not expect or ask for receipts. The scholarship on informal economy adopting the institutional theory framework collate the latter two pillars under the umbrella of informal institutions, as explained above when discussing the second wave of thought toward the informal economy in institutional theory.

**Figure 2:**
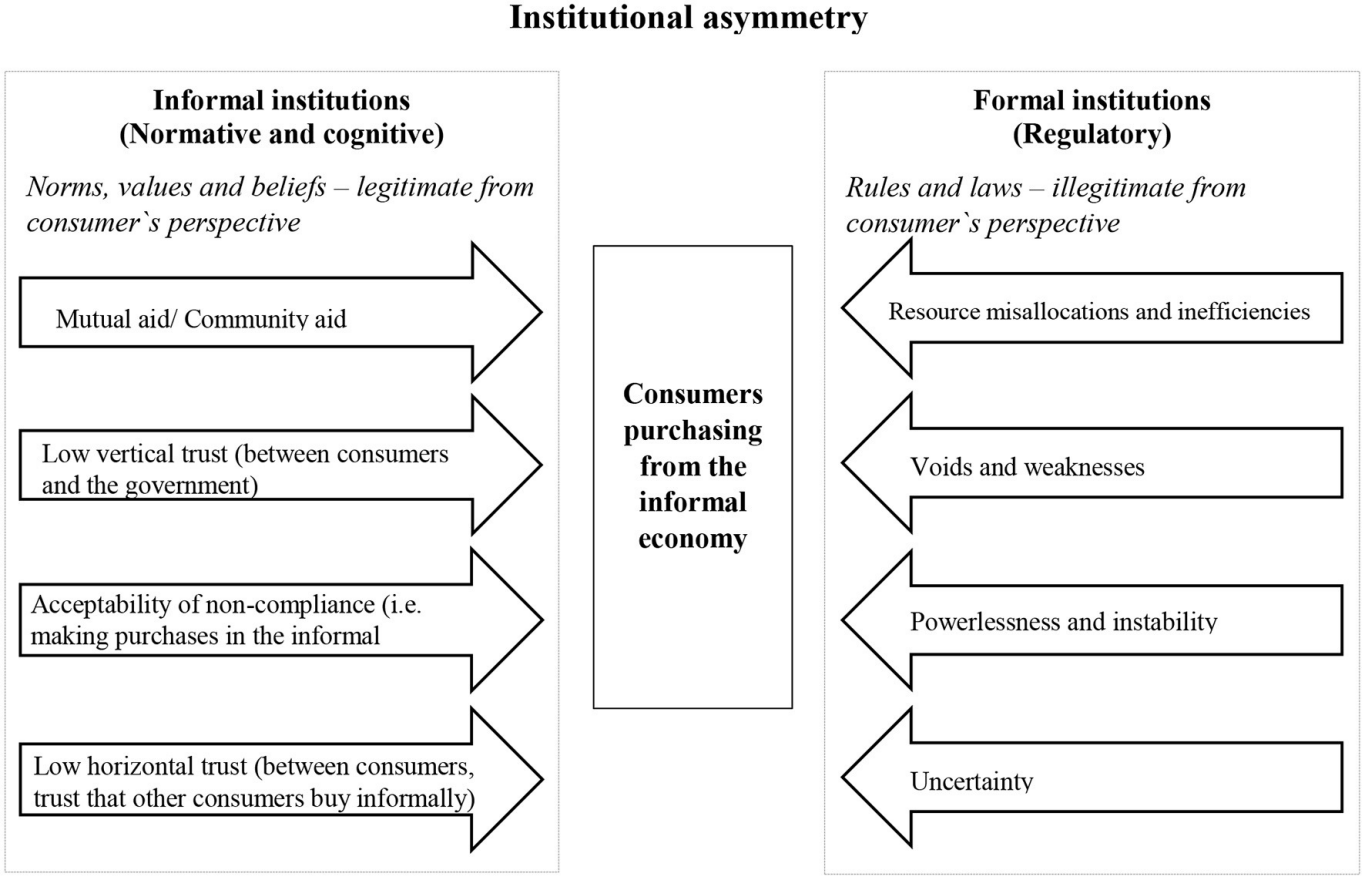
Consumer purchase model for testing the third wave of thought in institutional theory.

Scott (2008) argues that institutions exert pressure for compliance and adherence on individuals through the mechanism of isomorphism. Coercive pressure refers mainly to formal rules and regulations belonging of the formal institutions pillar. Normative pressure is the pressure to conform to wider societal expectations and is related to the normative pillar. Mimetic pressure is the pressure felt by citizens to act in ways that reflect the culturally supported shared understandings and common beliefs from a society and is related to cultural-cognitive pillar (Scott, 2008; Williams, 2017). Employing this view and adding the previous results found valid for the supply-side of the informal economy, as well as the scarce findings found so far on the demand-side, we propose the model displayed in Figure 2 to be tested in future research to obtain a fuller and better portrait of the motives that drive the consumer to the informal economy. This model enables a testing of the third wave of thought on the informal economy in institutional theory, a subject untouched so far by studies of consumers. The indicators and variables used to measure the formal and the informal institutions indicated in the arrows used for the supply-side of the informal economy can be used as a starting point to test this model (for a full review of these indicators and the results see Williams and Horodnic, 2020).

In addition, using cross-cultural and intercultural perspectives on the informal economy would help researchers discover the various facets of informality influences according to the values specific to different ethnic economies or different cultures, and adjust policy measures in accordance with cultural distances between countries (Light and Gold, 2000; Eskelinen, 2019). One issue of particular interest for the informal economy would be the way in which moral judgment development takes place across cultures (Gibbs et al., 2007), because it could account for differences in consumer behavior in different countries. Moreover, moral development and intercultural development have been found to be significantly related to each other (Endicotta et al., 2003), and could explain shifts from ethnocentric orientations to ethnorelative ones, thus helping researchers and policy makers explain and prevent informal economic behavior based on consumer ethnocentrism. This perspective could be enriched by taking into consideration differences related to the complex history of countries–such as civilizational competencies for Eastern and Central European countries who've been through a process of transition from a shortage or tensed centralized economy to a surplus one, or the ambivalence of social change, with triumph and trauma at the same time (Sztompka, 1993, 2000; Kornai, 2006).

In terms of methodological development future research should consider the inter- and trans-disciplinarity approaches forged into a complexity-system thinking perspective (sociology, psychology, marketing, behavioral economics, anthropology, in addition to economics perspective) (Rasanayagam, 2003; Dell'Anno and Schneider, 2009; Power and Mont, 2010; Diaz-Bone and Salais, 2011; Cortado, 2014). Although the anthropological perspective to the informal economy is not new, the voice of anthropologists remains rather shy and there are very few studies applying anthropological methods in order to observe, measure and explain informal consumer behavior. Similarly, experimental studies are not yet conducted to investigate consumer behavior in the informal economy. Moreover, studies usually apply a single or at the best a double disciplinary perspective, while a better understanding could be obtained through a complex approach, in which the main disciplines are brought together in order to obtain a clearer and wider view on consumer behavior in the informal economy.

A sub-stream of research could be dedicated to the analysis of informal purchasing and consumer behavior in the online environment, especially after crossing the COVID-19 pandemic and with the accelerated development of various mobile commerce apps. Studies on this subject did not touch the informal purchasing aspects, but generally analyzed consumer sentiment and behavioral intentions toward online shopping through mobile apps, showing that people's intentions to use delivery services and consumer habits in identifying online information are shaping their feelings and loyalty (Rowland, 2022; Watson, 2022; Zvarikova et al., 2022) and are influenced by perceived risk and trust consequences (Andronie et al., 2021; Smith and Machova, 2021)–all these being also important behavioral variables for the informal economy. A supplementary support for the investigation of the online behavior could be offered by artificial intelligence instruments, such as artificial neural network algorithms for the analysis of customer purchasing intentions or computer vision technologies for identifying user trends and patterns, including customer attitudes and feelings (Frajtova Michalikova et al., 2022; Hopkins, 2022; Nica et al., 2022).

Finally, and from increasing the practical implication of the consumer studies in the informal economy, a potent avenue is represented by applying the social marketing perspective for investigating consumer behavior in informal economy. According to the explanatory models used in social marketing, five stages should be considered for the consumer behavior: precontemplation, contemplation, decision, action and maintaining behavior (Dann, 2005; Carrigan et al., 2011; Wymer, 2011). Consumers of various informal products and services in a country are usually situated in different stages, depending on their knowledge, interest and attitudes toward social issues, including those created through the informal economy. From a marketing perspective, they should be targeted according to their stage, and both communication strategies used by practitioners and policy makers need to be tailored consequently. For pre-contemplating informal consumers, the focus has to be on triggering awareness and interest in order to shift their purchases from the informal to the formal economy – otherwise all messages would be ignored, and communication and policy measures wasted. For contemplating consumers, already aware about the existence and consequences of the informal economy, the focus is on weighting advantages and disadvantages for the behavioral shift taking into account that besides benefits and costs, consumers also consider social influences, formal market provision failures as well as perceived control over their choices and behavior. In the decision stage, for making the consumer choose the formal economy, the advantages have to be clearly higher in the balance, compared to disadvantages. In the action stage the change in behavior has to be portrayed as easy, through communication campaigns, and actually made easier, usually by offering support and nudges, financial or moral incentives–thus creating bridges for overcoming difficulties in the adoption process, increasing benefits, reducing costs, increasing perceived social pressure and control. Maintaining the wanted behavior (i.e., the purchases from the formal economy) needs continuous efforts in time, as long as necessary in order to remind consumers why they decided to shift and why it is good to continue in the formal economy, thus to enable new habits to be shaped and new norms to be built.

## Discussion and Conclusions

In this paper, we aimed to advance the knowledge on consumer behavior in the informal economy by providing a review of the previous studies on this topic. Surprisingly, despite the fact that more than 60% of the worldwide workforce have their main employment in the informal economy, displaying that informal economy represents a major sphere of production and consumption across the world, little research has been conducted on explaining consumer behavior in this realm. A systematic search revealed that <20 papers have been conducted in this field. Even more, the contemporary knowledge on the topic is based on studies conducted by a limited number of researchers, using a limited number of extensive databases (i.e., four databases) or small scale case studies. There are also major geographical areas where there has been no previous study on consumer behavior in the informal economy (e.g., the United States, Australia, Canada). Furthermore, the vast majority of studies use the same type of analysis (i.e., regression analysis) and methods such as experiments and anthropological studies are completely absent. Finally, these studies have been made rather from an economic or sociological perspective and published in journals from these fields, despite researchers warning that the literature and theories on consumer behavior are developed using empirical findings from formal markets and therefore, their applicability to other realms needs to be asserted with caution (Viswanathan et al., 2012).

We advanced the theory on consumer behavior in informal markets by providing a profile of the consumer in the informal economy and an inventory of the theoretical perspectives employed to explain the determinants that drive consumers to choose the informal economy. This revealed that rational economic actor theory (i.e., lower price) is not the solely motivational factor nor the most dominant. Furthermore, when analyzing from a temporal perspective, fewer consumers are declaring over time that their purchase from the informal economy is driven by financial gain. This therefore, is a call for practitioners to transcend the dominant view that consumers are driven by lower costs. The finding is that in many of the cases, consumers choose the informal economy due to the failures of formal market provision in terms of quality or speed of delivery. Therefore, practitioners should start to consider addressing these issues and adapting their offer such as to attract consumers from the informal economy to their goods and services. If they did so, they would also reach a social responsibility goal, joining their forces with the authorities in tackling the informal economy and reducing its negative effects on governments, workers and consumers.

In terms of methodology, this paper conducted for the first time a review of the research conducted in this area of the consumer studies. Finally, from a practical implications point of view, this paper enables the practitioners and the policy makers to better understand who the consumer is in the informal economy and why they make purchases from the informal economy. On the one hand, knowing the profile of the consumer helps both the practitioners and the policy makers to target and adopt marketing campaigns or educational campaigns to encourage the consumer to move to the formal economy. These campaigns could focus on improving the consumers' tax morale, as previous research showed that it is in all analyzed cases directly related with the likelihood of participation in informal economy. On the other hand, knowing which motives consumers use to explain their engagement in the informal economy, enables both the practitioners and policy makers to tailor their approaches to tackle the informal economy. For example, the findings showed that the consumer is not solely motivated by financial gains, therefore, in the cost-benefit ratio, the policy makers could focus to increase the benefits of formal economy instead of focusing on increasing the deterrence measures (i.e., the cost of such activity). This includes, for example service vouchers or lotteries of fiscal receipts organized in order to encourage the consumer to ask for a receipt when making purchases (Michalopoulos, 2017; European Commission, 2021), which will also reduce unintentional informal purchases. Similarly, practitioners can organize marketing campaigns focusing on the benefits of the formal goods and services such as guarantees, meeting the health and safety requirements and so on. However, as the findings show, consumers are not solely rational economic actors balancing the costs and the benefits of making purchases from the informal economy. Other motives such as the failures of the formal market provision drive the customers to purchase from the informal economy. Thus, addressing this issue by organizing trade fairs, developing sharing economy platforms, creating apps or other initiatives to enable contact between the customers and the providers can help in tackling informal economy and can be pursued by both private of public sector representatives. As for those motivated by social ends, measures can involve issues related with bureaucracy simplification or a fixed annual amount tax free for suppliers in order to allow the citizens to strengthen the social networks and improving the social capital (Valor and Papaoikonomou, 2016).

Based on the above contributions to the field and also based on an in-depth knowledge on the supply-side of the informal economy, we have here developed a rich research agenda to address the gaps in the consumer behavior literature and to understand how consumers behave in informal markets. As such, we propose in this paper valuable avenues for theoretical, methodological and practical implication perspectives. What is for certain, however, is that consumer research theory can no longer solely focus upon consumer behavior in the formal economy and ignore a large contemporary sphere of production and consumption.

If this paper stimulates scholars to conduct further quantitative and qualitative studies of consumer behavior in the informal economy, and perhaps explore further the use of institutional theory to explain how institutions shape consumer behavior, then it will have fulfilled one of its major intentions. If this then results in greater consideration of how to tackle consumption in the informal economy, and practitioners recognizing that consumers are driven to the informal economy for other motives beyond lower cost, and they may start to correct the failures of the formal market provision with their offer of goods and services and thus joining their forces with the public authorities in reducing this phenomenon, the paper will have fulfilled its wider intention.

## Data Availability Statement

The original contributions presented in the study are included in the article/supplementary material, further inquiries can be directed to the corresponding author.

## Author Contributions

All authors listed have made a substantial, direct, and intellectual contribution to the work and approved it for publication.

## Funding

This work was supported by a grant of the Ministry of Research, Innovation and Digitization, CNCS/CCCDI–UEFISCDI, project number PN-III-P1-1.1-TE-2019-0229, within PNCDI III.

## Conflict of Interest

The authors declare that the research was conducted in the absence of any commercial or financial relationships that could be construed as a potential conflict of interest.

## Publisher's Note

All claims expressed in this article are solely those of the authors and do not necessarily represent those of their affiliated organizations, or those of the publisher, the editors and the reviewers. Any product that may be evaluated in this article, or claim that may be made by its manufacturer, is not guaranteed or endorsed by the publisher.
